# Percutaneous osteosynthesis of Galeazzi fracture-dislocation

**DOI:** 10.4103/0019-5413.67121

**Published:** 2010

**Authors:** Wasudeo M Gadegone, Yogesh Salphale, DS Magarkar

**Affiliations:** Department of Orthopedics and Traumatology, Chandrapur Multispecialty Hospital, Mul Road, Chandrapur - 442 401, India

**Keywords:** Elastic nail, intramedullary nail, Galeazzi fracture dislocation

## Abstract

**Background:**

A Galeazzi fracture is defined as a fracture of the radius associated with dislocation of the distal radio-ulnar joint (DRUJ). The conventional surgical technique of nailing does not give enough stability and open reduction, internal fixation with the plate is associated with numerous complications. The stacked nailing for the management of these injuries provides adequate stability, maintains the relationship of the DRUJ and promotes uneventful union by closed technique. The purpose of this study is to evaluate the results of simple, user-friendly, low cost elastic stacked nailing for the management of Galeazzi fracture dislocation.

**Materials and Methods:**

We treated 22 young adults with fresh Galeazzi fracture-dislocation of the forearm, from January 2004 to January 2008, by percutaneous fixation of fracture by stacked elastic nailing at our institute. There were 19 males and three females and the age group ranged from 20-56 years (average 35 years). Surgery was performed within 48 to 72 hours under the guidance of image intensifier. Medullary cavity was filled with two elastic titanium nails having unequal lengths and diameter. One nail acts as a reduction nail and the other acts as a stabilizing nail. The results were evaluated using Mikic criteria based on union, alignment, relationship of the DRUJ, and movements at the inferior radio ulnar joint, elbow and wrist.

**Results:**

In six cases, following radiological union, nails in the radius were extracted between six to nine months after operation because of discomfort complained by the patient at site of insertion. After one year follow-up, 18 patients had excellent, four had fair results.

**Conclusion:**

Closed reduction and internal fixation of Galeazzi fracture by two elastic rods re-establishes the normal relationship of the fractured fragments and the DRUJ without repair of the ligaments. The stability is achieved by the flexibility and elasticity of the nails, crowding of the medullary canal and anchorage they gain in the radial diaphysis. Elastic nailing can produce excellent clinical results for Galeazzi fracture-dislocation. It has the advantages of technical simplicity, minimal cost, user-friendly instrumentation, and a short learning curve.

## INTRODUCTION

The fracture of the radius and dislocation of the distal radio-ulnar joint (DRUJ), described by Sir Astley Cooper in 1824, is a very unstable and a rare injury.^1^ Galeazzi, (1934), described fractures of the middle and distal third of the radius associated with instability of the DRUJ. Galeazzi fracture patterns reportedly account for six to seven per cent of all forearm fractures in adults.^2^ This type of lesion is characterized by its unstable nature; a high index of suspicion should be maintained by the surgeon, and a thorough examination for instability of the DRUJ must be conducted.^3^ A high failure rate with conservative management by closed reduction in adults has been reported.^4^–^6^ Use of Rush pins, Kirschner wires, triangular nails and square nails has been reported with varying success.^7^–^9^

Plating of the fracture of the radius is most suitable in these cases and if correctly done it provides rigid internal fixation of the bone and stable reduction of the DRUJ in most cases.^10^,^11^ Although plate is commonly used to fix Galeazzi fractures, however, soft tissue dissection entails various complications such as loss of fixation, sepsis, delayed union or non-union, refracture after plate removal, and risk of intraoperative nerve injuries. Nailing is performed with minimum instrumentation, respecting the biomechanical principles and achieving an eventually good functional outcome comparable to the existing standard surgical technique.

The purpose of this article is to evaluate a simple, low-cost, elastic stacked intra medullary nailing for the management of Galeazzi fracture dislocation. and the stability it can provide in this inherently unstable injury.

## MATERIALS AND METHODS

Twenty two patients with fresh Galeazzi fracture dislocations treated from January 2004 to January 2008 at our institute constitute the study. The mean age of a patients was 35 years (range 20-56 years). Most fractures occurred at the junction of the middle and distal third of the shaft of the radius [Table 1]; 11 fractures were transverse, nine oblique, and two had comminution. Three had associated injuries in the form of fractures of the pelvis and fracture of the metacarpals.

**Table 1:** Site of the fractures and associated injuries of distal radio ulnar joint

Fracture site	n	Associated injuries of distal radioulnar joint		
		Dislocation	Subluxation	Frx of ulnar styloid process
Middle third	9	5	2	2
Junction of middle and lower third	13	9	3	1
Total	22	14	5	3

n = Number, frx = Fracture

### Operative procedure

**Figure 1:**
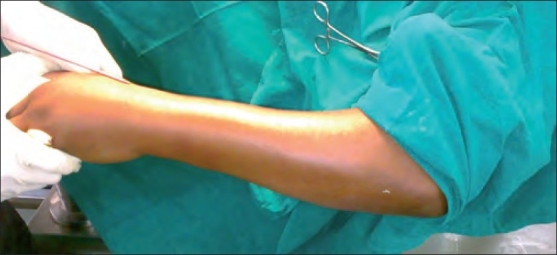
Clinical photograph showing the reduction maneuver and introduction of the nail

Two fractures were Grade I open injuries as per Gustilo and Anderson’s classification. Nineteen fractures were operated under brachial block and the rest three were given general anesthesia. For open fractures, reduction was undertaken after debridement of the wound. All fractures were operated under tourniquet. The fracture was reduced under C-arm guidance with manual traction exerted on the hand by the assistant after abducting the arm and flexing the elbow with the help of elbow attachment [Figure 1]. Each of the 22 patients was treated by two elastic nails - one for reduction and the other for stabilization of fracture.

A 2-3 mm pre bent elastic titanium nail (Yogeshwar Implants, Thane, India) was introduced through the styloid process of the radius utilizing a stab incision and negotiated till it reached the subchondral bone of radial head. This acts and behaves as a reduction nail. Another nail of different size and length was then passed across the fracture site into the medullary canal till further negotiation was not possible and the stability of the construct was assured by manual rotation of the construct under C arm. The forearm was then rotated to assess for any DRUJ instability. The entry site was dressed and a compression dressing was given. We had to stitch the entry wound in only two cases during the early part of this study.

If the DRUJ was found to be stable in supination, splintage in a long arm splint was given in supination for six weeks. If the DRUJ was reducible in supination but unstable, stabilization was achieved by placing 2 mm kirschner wires (K-wires) from the ulna into the radius (*n*=2), just proximal to the articular surface. In a case of fracture of the base of ulnar styloid, plaster immobilization was continued for a period of six weeks.

**Figure 2:**
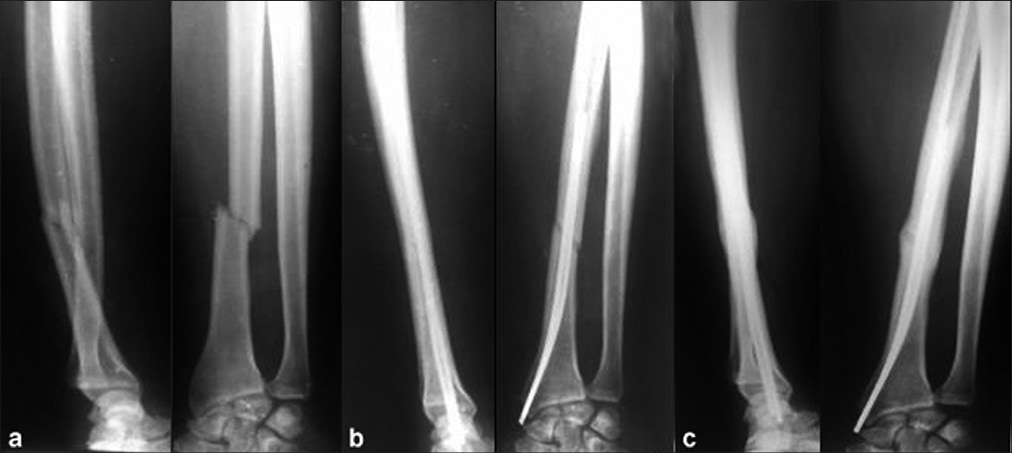
Lateral and anteroposterior X-ray of right forearm showing (a) a Galeazzi fracture dislocation treated with closed elastic stacked intramedullary nailing (b) early union and good alignment at 12 weeks followup, (c) progressing to sound fracture union and well aligned DRUJ at 12 months follow-up

Radiographs in anteroposterior and lateral views were evaluated for healing of the fracture, reduction and alignment of the radius, and the congruity of the surfaces of the DRUJ. Radiographs were taken at four weeks, eight weeks, twelve weeks and between 6-12 months at three monthly interval [Figure 2]. The comparison radiographs of the opposite wrist were made for measurement if radial shortening was suspected on clinical examination. At four weeks, radiographs were obtained to check the alignment and reduction of the radius and DRUJ. At six weeks, cast was removed, radiographs obtained, and physical therapy for elbow, wrist, and digits was initiated. Pins passed for the stability at the DRUJ were removed, if present after four weeks and the cast in full supination is continued for further two weeks. The wound and the fractures healed uneventfully in both the cases with grade I compound fractures.

Results were classified according to Mikic’s criteria.^15^ They were rated as excellent, fair and poor. The excellent result is one in which there is union, perfect alignment, no subluxation or dislocation of the DRUJ, no limitation of supination / pronation and the elbow and wrist joint motion. A fair result is one in which there is delayed union, minimum mal alignment, minimal restriction of supination or pronation and the motion at elbow and wrist. The result is rated as poor if there is a nonunion, DRUJ and gross restriction of supination and pronation and motion at the elbow and wrist joint [Table 2].

**Table 2:** Mikic criteria of functional evaluation

Criteria	Excellent	Fair	Poor
Union	Yes	Delayed	Non union
Alignment	Perfect	Minimal	Disturbed
Length	No discrepancy	Minimal shortening	Gross shortening
DRUJ	No subluxation	Subluxation	Dislocation
ROM at			
(1) Elbow and wrist	Full	Some degree of restriction	Gross restriction
(2) Pronation and supination	Full	Less than 45° of restriction	More than 45° of restriction

## RESULTS

The average time taken for the surgery was 30 minutes (average 25-40 min). The follow-up period in our series was twelve months to 18 months with an average of 14 months. Our results were excellent in 18 cases and fair in four cases as per Mikic criteria.

We had one case of superficial infection at the site of the nail entry which responded well to regular dressings and healed up uneventfully. In four cases subluxation of the DRUJ with restricted pronation and supination was noticed during the follow-up period. In two cases it was noted after the cast removal, although it had a stable fixation and a normal alignment in the immediate post operative period. There was loosening of the cast in these two cases in the early part of the study. In the remaining two cases there was a progressive collapse of the radial fracture and the subluxation of the DRUJ was noticed three months after the surgery. All these four cases healed uneventfully and the patients regained good functional range of movement without necessitating further surgery. Complications were recorded in these four patients with fair results.

No patient had delayed union or nonunion and nerve injury. Six patients reported discomfort at site of insertion of nail hence the nail was extracted in less than 9 months. The earliest time period necessitating nail removal was after 6 months of operation, following union of the fracture There was no difference in clinical outcome of the patient with fracture of the ulnar styloid process.

## DISCUSSION

Galeazzi fractures are inherently unstable injuries. They are estimated to account for six to seven per cent of all forearm fracture in adults. The brachioradialis muscle and the extensors and abductors of the thumb tend to shorten the radius while the pronator quadratus rotates the distal fragment towards the ulna.^12^ Due to the strong muscle forces acting at the wrist and the likelihood of re-dislocation of the DRUJ. It often gives unsatisfactory to poor results, if managed conservatively. Even rigid osteosynthesis of the radius does not guarantee stable reduction of the DRUJ^4^,^13^,^14^. A high index of suspicion, early recognition, and acute treatment of DRUJ instability will avoid chronic problems in this complex injury.^3^

The radius is a curved bone with its concavity towards the ulna. The medullary canal of the radius is funnel-shaped in the distal third, and curved and narrow in the middle third^15^ making it somewhat unsuitable of intramedullary fixation. If the Kuntscher principle of filling the canal is to be followed, a straight rigid nail can not meet all the necessary requirements

In order to address the issue, a pre bent triangular metal nail sufficiently elastic and resilient while it traverses the canal from the point of insertion, but rigid enough to withstand the torsional, rotational, and angulatory forces while progressing to union was devised which gave fewer instances of malunion and non-union compared with other methods of open reduction and internal fixation.^7^ The flexible Rush pins^8^ follow and maintain the radial curve, and impart stability to ensure a good fixation of the fragments.

Internal fixation by square nail in Galeazzi fracture of the distal third of the radius not only permits rotatory motion at the fracture site, but also substantial lateral movement, which may predispose to delayed union and non union. It might lead to subsequent instability at the DRUJ. The end-of-the-rush nail acts as a potential irritant to the tendons around the wrist, necessitating early removal.

Open reduction and rigid internal fixation of the fracture of the radius re-establishes the normal relationship of DRUJ without repair of the ligaments. Fixation with a plate and screw is superior to square nail fixation in these fracture.^10^ Open reduction and internal fixation by plate and screws is widely used all over the world with variable results. In the series of 21 Galeazzi fractures treated by ASIF plating, Dodge and Cady^17^ found union in all the fractures; one patient had troublesome loss of motion and one had noticeable shortening. Mikic^15^ in his series of 125 patients had fourteen children and eighty-six adults with classic Galeazzi lesion. Twenty patients had the fracture of both the bones of the forearm which involved dislocation of the distal radio ulnar joint, Forty-two patients with classic injuries were managed by open reduction of the radius by various methods such as Rush Nails, Kuntschner Nails, K wires and wire loops with varying results. In 13 of these, the fractures were plated with generally satisfactory results (six excellent, four fair and two poor whereas the result of one patient was not mentioned). The cases treated conservatively, gave a poor result.

Early compression-plating with a six-hole plate will give satisfactory results for most fractures. Grafting is usually not necessary, and four weeks of immobilization in neutral pronation-supination or mild supination is adequate. Resection of the distal end of the ulna or temporary fixation of the distal radio-ulnar joint with a pin through the radius and ulna is rarely, if ever, required after compression plating of a fresh unstable fracture.^2^

The radius is usually bowed in two planes- one in the frontal plane having a lateral convexity and the other in the saggital plane with posterior convexity.^16^ This anatomical configuration makes it difficult to obtain adequate compression across the fracture surfaces. In the published series of diaphyseal fractures in the forearm that were treated by plating, complications such as loss of fixation in 0-5%, sepsis in 3-9%, delayed union or non-union in 2 -13%, and refracture after plate removal in 1-3% have been documented. lntraoperative nerve injuries occurred with an unexpectedly high frequency^7^. Reported incidence of transient dorsal interosseous nerve palsy is 7-10% of all patients with a radial fracture that was treated with an ASIF plate.^17^,^18^

In our series, no nerve injuries were found. A single medullary nail within the radius has no inherent stability to withstand the muscle forces. Therefore a second nail of a shorter length, engaging into the narrowest part of the radius prevents the rotation of the fracture fragments and imparts fixation on the principle of three-point fixation and Kuntschner’s principle of crowding of the medullary canal. Similarly, Hackethal who had proposed nailing for the forearm fractures and achieved excellent results based the utility of his nailing on the four premises, especially the jamming of the nails in the cortical window, jamming the waist of the medullary cavity, spreading the bunch of nails in the metaphysis and filling up the conus of the medullary cavity with short nails.^19^

An above elbow plaster cast in full supination avoids opening and repair of the inferior radioulnar joint complex.^5^,^16^ The avulsion of the ulnar styloid process is equivalent to fibrocartilage disc rupture and it has been reported that fractures of the ulnar styloid process in 60 percent resulted in nonunion of the styloid process^20^ but that function was not impaired by the union or nonunion of the ulnar styloid.^21^ We agree that nonunion of the styloid process does not affect the end results, as noticed in three cases in this series.

The use of elastic stable intramedullary nailing for displaced and unstable fractures of the radius and ulna in children is a well established method,^22^,^23^ but there is paucity of literature in the management of adult Galeazzi fractures. We analyzed the literature and reviewed and compared our results [Table 3]. We agree that a high index of suspicion should be maintained by the surgeon, and a thorough examination for instability of the DRUJ must be conducted to achieve good results.^24^,^25^ Open revision, repair of the triangular fibrocartilage complex and immobilization of the wrist are not necessary if anatomic reduction of the joint is obtained by indirect means.^26^ We suggest aggressive surgical treatment in adults addressing bone and soft tissue injuries. We feel that the four cases in our series which had subluxation at followup could have been prevented had the ulno-radial transfixation with a Kirschner wire performed.

**Table 3:** Comparative analysis with the previous studies

Author	Adult patients	Osteosynthesis	Results		
			E/G	F	P
Mikic Z D (1975) 1	13	Plating	6*	4	2
Dodge, Cady (1972)	21	Plating	19	-	2
Strehle J, Gerber C (1993)	19	Plating	16	3	-
Mohan K *et al*. (1988)	11	Nailing	4	7	-
	29	Plating	28	-	1
Present series	22	Nailing	18	4	-

E/G = Excellent to good, F = Fair, P = Poor

* = Data unavailable for 1 patient

Elastic stable intramedullary nailing of the radius is technically simple, cost effective with user friendly instrumentation, short learning curve and a cosmetic method with minimal risk of infection, delayed union, or tendon rupture, obeying and respecting the integrity of the soft tissues around the injured site. It provides anatomical reduction with preservation of normal curves of the radius and provides a stable fixation in both angulatory and rotational planes. The stability is achieved by the flexibility and elasticity of the nails and crowding of the medullary canal and anchorage they gain in the radial diaphysis. Our method of percutaneous insertion of the nail through the styloid process avoids injury to tendinous and the neurovascular structures of the lower end radius. This study aims to address the alternative surgical modality available for the management of these difficult injuries.
